# Life-Threatening Hypertriglyceridemia in Diabetic Ketoacidosis Successfully Managed With Therapeutic Plasma Exchange: A Case Report and Review of Literature

**DOI:** 10.7759/cureus.115264

**Published:** 2026-08-26

**Authors:** Zazeba Hossain, Asif Ahmed, Mahzabeen Moushumi, Mohammad Salim, Quazi Tarikul Islam

**Affiliations:** 1 Internal Medicine, Popular Medical College Hospital, Dhaka, BGD; 2 Critical Care Medicine, Popular Medical College Hospital, Dhaka, BGD; 3 Critical Care Medicine, Dhaka Medical College Hospital, Dhaka, BGD

**Keywords:** aki, dka, hypertriglyceridemia, multiorgan failure, plasmapheresis, pneumonia

## Abstract

Extremely severe hypertriglyceridemia associated with diabetic ketoacidosis (DKA), shock, cerebral edema, acute kidney injury (AKI), and pneumonia represents a rare but life-threatening clinical emergency, particularly when occurring as the initial presentation of previously undiagnosed diabetes mellitus (DM). In such critically ill patients, prompt management is challenging because clear evidence-based guidelines regarding the role and timing of therapeutic plasma exchange (TPE) remain limited. To our knowledge, this is the first reported case from Bangladesh describing severe combined hyperlipidemia with DKA as the initial manifestation of DM requiring urgent TPE.

A 15-year-old girl presented with altered consciousness and hemodynamic instability. She underwent immediate intubation upon admission due to severe metabolic decompensation. Further evaluation revealed DKA with extremely severe hypertriglyceridemia associated with combined hyperlipidemia. On the second day of hospitalization, her condition was further complicated by pneumonia and AKI. Despite standard intensive management, including insulin therapy, fluid resuscitation, and supportive care, no significant overall clinical improvement was observed. Considering the progressive deterioration and risk of fatal complications, TPE was initiated on day 3. Following a single session of TPE, her clinical status improved dramatically, with rapid hemodynamic stabilization and metabolic recovery, allowing successful weaning from ventilatory support within 24 hours.

This case highlights that TPE may be a valuable adjunctive treatment in carefully selected patients with DKA complicated by extremely severe hypertriglyceridemia and multiorgan dysfunction when conventional therapy fails to produce adequate clinical improvement. We also review previously reported cases and discuss the potential role of TPE in managing this rare but life-threatening condition. As plasmapheresis remains costly and less accessible in resource-limited settings like Bangladesh, greater clinical awareness and further evidence are needed to guide future management recommendations for similar critically ill patients.

## Introduction

The potentially fatal metabolic condition known as diabetic ketoacidosis (DKA) is characterized by uncontrolled hyperglycemia, metabolic acidosis, and ketosis, which is a common and dangerous complication of type 1 diabetes mellitus (DM) [1]. According to a recent study, the pooled risk of DKA at type 1 diabetes diagnosis in 13 high-income nations was 29.9% from 2006 to 2016, whereas in Asian countries like Malaysia, reports were much higher (54-75%) [2,3]. Although Bangladesh exhibits one of the highest prevalence rates of DM within the Southeast Asian region (12.3 % in 20-79 years), there is no such database for type 1 DM patients [4]. Hypertriglyceridemia is observed in approximately 30-50% of DKA cases, which is caused by insulin deficiency by two main actions: first, increased lipolysis releases free fatty acids that speed up the liver's production of very-low-density lipoprotein (VLDL). Secondly, peripheral tissues' decreased lipoprotein lipase (LPL) activity slows down the removal of chylomicrons and VLDL from the blood. There is an accumulation of triglycerides in the bloodstream as a result of this dual action of increased synthesis and decreased clearance [5]. DKA can lead to serious complications like hypoglycemia, hypokalemia, cerebral edema, rhabdomyolysis, pancreatitis, respiratory failure, etc. [6]. According to the Endocrine Society classification of hypertriglyceridemia, severe and very severe hypertriglyceridemia is graded above 1000 and 2000 mg/dL, respectively. Severe hypertriglyceridemia in the background of DKA is associated with significant morbidity and mortality. Therefore, prompt treatment is warranted to avoid complications like acute pancreatitis and thromboembolism [7]. Along with lifestyle modification, dietary counselling, physical activity, and weight reduction, adding drugs like fibrates, niacin, and omega-3 fatty acids alone or in combination with statins is recommended to treat severe or very severe triglyceridemia [7]. The role of plasmapheresis as an add-on management is understudied and underreported. Here, we report a case with a >14000 triglyceride level complicated by DKA and newly diagnosed DM with sepsis managed with plasmapheresis, which led to rapid stabilization of the patient.

## Case presentation

**Figure 1:**
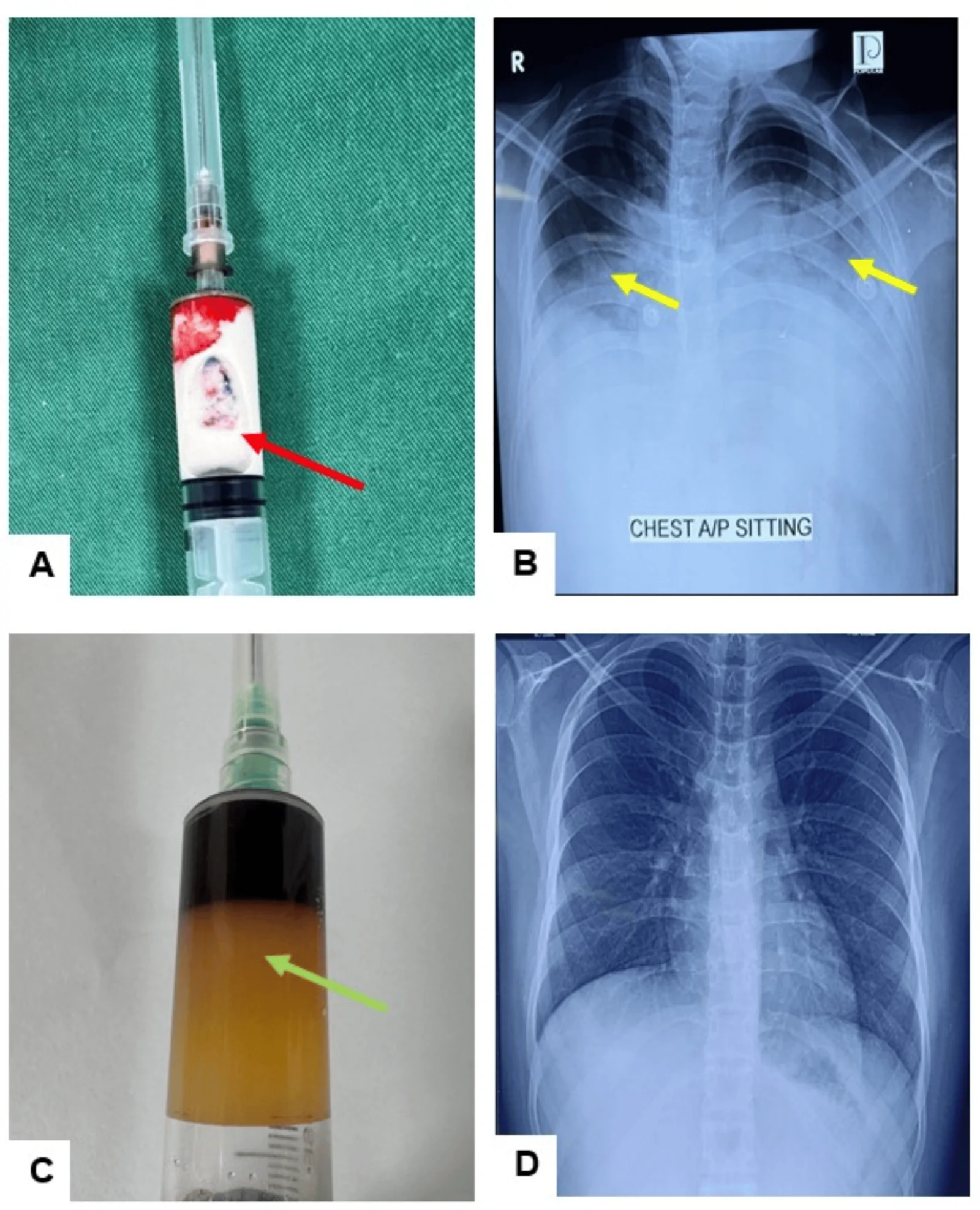
Clinical and radiographic findings before and after plasmapheresis (A) shows the patient's serum on the day of admission; the red arrow marks the white, thick, lipemic serum. (B) shows the admission chest radiograph with bilateral pulmonary Infiltrations (yellow arrow). (C) shows her serum after plasmapheresis (green arrow). (D) almost normal chest radiograph just before discharge

A 15-year-old girl with no previously known comorbidities was admitted into our hospital’s critical care unit through the emergency department with complaints of altered level of consciousness for nine hours, shortness of breath and chest pain for 14 hours, and diffuse abdominal pain for two days. Apart from excessive thirst, there were no other features suggestive of DM. Her father had type 2 DM, and her mother had a history of gestational DM (GDM) and was receiving lipid-lowering drugs due to hypercholesterolemia. All of her siblings were in good health and had no significant history. On admission, she was unconscious with a Glasgow Coma Scale (GCS) score of 7/15 (E1M4V2). There was no neck rigidity or focal neurological deficit, and fundoscopic examination was normal. Her heart rate was 142 beats/min (regular), temperature was 98°F, blood pressure was nonrecordable, and respiratory rate was 40 breaths/min with Kussmaul breathing. Oxygen saturation was 90% on room air, improving to 98% with 5 L/min oxygen via nasal cannula. Chest examination revealed bilateral coarse crepitations (more on the right side). The abdomen was distended and diffusely tender. Her urine output was inadequate. Blood drawn for laboratory investigation appeared milky white in color (Figure 1A). Her random blood glucose was 24 mmol/L. Arterial blood analysis (ABG) revealed severe metabolic acidosis and respiratory alkalosis (Table 1). An urgent CT brain was done, which was unremarkable. Liver function (serum (S) bilirubin, prothrombin time (PT), albumin, alanine aminotransferase (ALT), and aspartate aminotransferase (AST)), lipase, urea, thyroid profile, toxicology profile, and echocardiography were all within normal limits. Bedside ultrasonography did not reveal pancreatic swelling, and CT of the abdomen could not be performed because of her hemodynamic instability.

**Figure 2:**
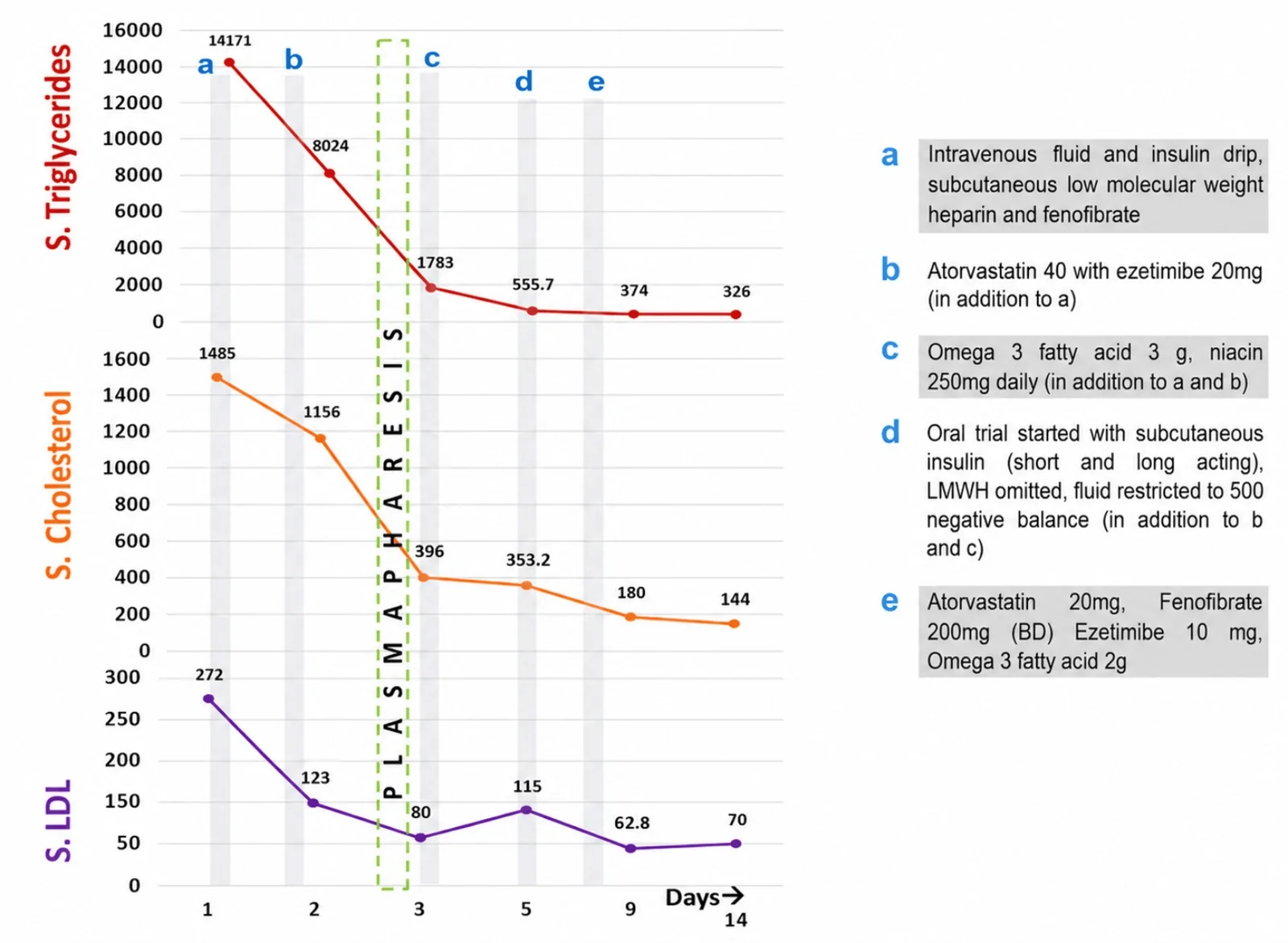
Graphical representation of triglyceride level (in mg/dL, along ordinate) during hospital stay (along abscissa) with treatment The green dotted area represents plasmapheresis on day 3

She was started on noradrenaline (.5 mcg/kg/min) after failed resuscitation with crystalloid fluid (1L). However, she remained further hemodynamically unstable and subsequently developed worsening hypoxemia, requiring mechanical ventilation. She further developed bilateral rhonchi, and therefore, a single dose of intravenous hydrocortisone 100 mg was given with escalation of insulin dose to manage the upsurge of sugar considering the risk-benefit ratio. She was kept nil per oral except medications via nasogastric tube and was managed with intravenous fluid, insulin drip according to protocol, subcutaneous low molecular weight heparin (40 IU/day), nebulization with bronchodilator, and injectable proton pump inhibitors and fenofibrate (200 mg, twice daily). Atorvastatin 40 mg, ezetimibe 20 mg, omega-3 fatty acid 3 g, and niacin 250 mg were also added gradually during ICU stay (Figure 2). Her level of consciousness did not improve; rather, on her second day of hospitalization, she developed fever, worsening pedal edema, and ascites. Respiratory crepitations also worsened as she developed severe pneumonia (Figure 1B). Her blood, urine, and tracheal aspirate cultures revealed no growth, but her endotracheal tube tip for culture yielded *Staphylococcus hemolyticus*. She also had high infective markers (Table 1). Thereafter, broad-spectrum antibiotics (teicoplanin and meropenem) were administered.

**Table 1:** Laboratory parameter of the patient after admission and before discharge ACR: albumin creatinine ratio; LDL: low-density lipoprotein; LDH: lactate dehydrogenase; GAD: glutamic acid decarboxylase; IAA: insulin autoantibody; Zn T8: zinc transporter 8; ISA: islet cell antibody; *done 10 days after discharge

Lab parameter (units)	Normal range	After admission	Before discharge
Hemoglobin (g/L)	120-150	84	110
Total white blood cells (x10^9^/L)	4-10	36.3	8.8
Neutrophil (%)	40-80	75	50
Platelet (x10^9^/L)	150-410	250	300
pH	7.35-7.45	6.97	7.4
HCO3 (mmol/L)	22-26	7.8	23
pCO2 (Kpa)	4.7-6.0	3.9	5
S. osmolality (mOsm/kg)	275-295	268	280
Urine ketones	Nil	+++	-
Urine protein	Nil	+	+
Urine ACR (mg/mmol)	<3.4	-	40.8
S. sodium (mmol/L)	135-145	117	139
S. potassium (mmol/L)	3.5-4.5	3.1	4.0
S. magnesium (mmol/L)	0.6-0.95	0.5	0.9
HbA1c (%)	<6.5	12.7	-
S. vitamin D (nmol/L)	50-150	8.7	44.9
S triglyceride (mg/dL)	<150	14171	326
S. cholesterol (mg/dL)	<200	1485	144
LDL (U/L)	<100	272	70
S creatinine (µmol/L)	44.2-79.6	41.5	88.4
Serum lipase (U/L)	23-300	187	-
Serum albumin (g/L)	35-52	34	26
S uric acid (µmol/L)	154.6–356.9	505.6	357
D-dimer (µg/mL)	<0.5	4	.05
Lactate (mmol/L)	0.67–2.11	3.00	<0.22
Procalcitonin (µg/L)	<0.05	54.14	1.0
LDH (U/L)	135-214	882	200
Serum insulin level (pmol/L)	20.8–118.1	126.4	120*
Serum C-peptide level (nmol/L)	0.26–1.72	0.52	1.0*
Anti GAD-65 (IU/mL)	<17	-	2.7
IAA (IU/mL)	<20	-	8.54
ZnT8 antibody (U/mL)	<10	-	<15
ISA (U/mL)	<28	-	8.61

Despite treatment, there was minimal improvement in her blood lipid levels, acidosis (triglycerides 8024 mg/dL, cholesterol 1156 mg/dL, LDL 123 mg/dL, pH 7.31, and HCO3 18.9 mmol/L), and there was persistent ketonuria (++) and proteinuria (++). Therefore, the decision of therapeutic plasmapheresis was taken by the attending physician on the third day of admission. After a single session of plasmapheresis, her respiratory distress, consciousness, abdominal distension, and urine output improved. Figure 1C shows her serum after plasmapheresis. Her septic markers also came down (total white cell count: 13.5 x10^9^/L; S lactate: 1.08 mmol/L; C-reactive protein (CRP): 29.02 mg/L; procalcitonin: 6.28 μg/L; and D dimer: 0.89 µg/mL). She was successfully extubated the day following therapeutic plasma exchange (TPE) (day 4). Thereafter, she was commenced on an oral diabetic, low-fat, and renal diet. Insulin infusion was transitioned to a basal-bolus regimen. The only complication after TPE was hypoalbuminemia (2.6 mg/dL), which was managed by injectable albumin (100 mL of 25%, for three days). There was a drastic improvement in her serum lipid levels (Figure 2). She was stepped down to the medicine ward on the fifth day of admission and discharged on day 14 with a basal-bolus subcutaneous insulin regimen, atorvastatin 20 mg, fenofibrate 200 mg (BD), ezetimibe 10 mg, and omega-3 fatty acid 2g/day (Figure 2). During her inpatient period, her respiratory symptoms resolved, and her chest radiograph also improved (Figure 1D). She was completely weaned off from oxygen support by day 10. Her diabetic nephropathy was managed with oral ramipril (2.5 mg, BD) and finerenone (10 mg, OD). FibroScan of the liver excluded steatosis. At follow-up 10 days after discharge, her insulin and C-peptide levels were within normal limits (Table 1). Her fasting lipid profile had returned to normal, and no further complications were identified.

## Discussion

After reviewing similar literature, we found several case reports of DKA with very high (>500mg/dL) or severe (>1000 mg/dL) hypertriglyceridemia. The highest reported triglyceride was 40,176 mg/dL, described by Kravetz et al. [8]. Among these cases, only seven patients went through TPE as a treatment modality, where time to normalization of hypertriglyceridemia was two to 16 days (Table 2). All of the seven cases we reviewed presented or eventually developed pancreatitis, as expected in this clinical setting. However, in our case, the pancreas was spared. Four out of the seven cases were admitted to the critical care unit, and there were no deaths reported. In these reports, the majority were newly diagnosed T1DM. In addition, associations with GDM [9] and type 2 DM have also been reported [10,11]. Most reported patients were female, which is similar to our case. Other complications reported in the literature were acute kidney injury (AKI) [12,13], acute hepatitis [8], and fatty liver [9,13]. Rare associations were type IX glycogen storage disease (GSD) [14] and altered apolipoprotein status [11].

**Table 2:** Review of similar case reports with hypertriglyceridemia and DKA managed with TPE DKA: diabetic ketoacidosis; TPE: therapeutic plasma exchange; DM: diabetes mellitus; AKI: acute kidney injury; GSD: glycogen storage disease; GAD: glutamic acid decarboxylase; NPO: nothing per orem; SC: subcutaneous; IV: intravenous; ICU: intensive care unit

Age, gender (country)	Association	Peak triglyceride level (mg/dl)	Complication/notable findings	Treatment given	ICU admission	Time to normalize
10-year-old, female (USA)	T1DM	16334	AKI, pancreatitis	Fluid resuscitation, insulin drip, plasmapheresis x1 (3936 ↓), fenofibrate	No	2 days [12]
16-year-old male (USA)	T1DM	40176	Pancreatitis, hepatitis	Fluid resuscitation, insulin drip, morphine, plasmapheresis x2 (>1500↓,382↓)	No	>2 days [8]
38-year-old female (USA)	GDM	829	Pancreatitis, fatty liver	Fluid resuscitation, insulin drip, gemfibrozil, heparin, morphine, plasmapheresis x2, glyburide	No	8 days [9]
37-year-old, male (Italy)	DM	7000	Pancreatitis	Plasmapheresis x1 (6020), fluid resuscitation, insulin	Yes	2 days [10]
17-year-old male (Turkey)	Consanguineous issue, idiopathic T1DM	10700	Type IX GSD, pancreatitis	Fluid resuscitation, Insulin drip, plasmapheresis x1 (7650 ↓)	Yes	16 days [14]
51-year-old male (USA)	T2DM, occasional alcohol intake	>10000	Pancreatitis, altered apolipoprotein profile	Fluid resuscitation, Insulin drip, potassium replacement, plasmapheresis x1 (9577 ↓), fenofibrate	Yes	>1 week [11]
16-year-old female (India)	New onset DM T1A (anti-GAD positive), fatty liver (newly detected)	2870	Necrotizing pancreatitis, AKI, mild hyponatremia	Fluid resuscitation, insulin drip, NPO, analgesia IV, antiemetic IV, plasmapheresis x3 (1472↓), SC insulin ( both long and short acting), statin, fenofibrate, saroglitazar, low-dose prednisolone	Yes	15 days [13]

Our patient tested negative for diabetes-related autoantibodies. Her insulin and C-peptide levels were normal. Genetic testing (such as HNF1A, GCK, and HNF4A mutations) to exclude maturity-onset diabetes of the young (MODY) could not be performed due to financial constraints and the unavailability of testing. Severe hyponatremia and hypovitaminosis D in our patient may be explained by analytical interference from high triglyceride levels. The combination of elevated urinary albumin-to-creatinine ratio (ACR) and markedly elevated HbA1c suggested that diabetes had likely been present for several years [15]. Our case may fall into type IV phenotype (familial combined hyperlipidemia) according to the WHO classification [16], because she had severe hypertriglyceridemia accompanied by elevated total cholesterol and LDL cholesterol. However, quantification of ApoB level and other non-HDL cholesterol levels was not possible. Her mother has hypercholesterolemia, except that family history was otherwise unremarkable. Quantification of apolipoproteins by immunoassay and detection of genetic mutation for glycogen disease could not be done, which could further clarify her background diagnosis.

TPE is an extracorporeal blood purification technique in which plasma is separated from whole blood and replaced with albumin, plasma, or other replacement fluids. It is recommended as second-line therapy for familial hypercholesterolemia. It is also used in severe hypertriglyceridemic pancreatitis and selected cases requiring relapse prevention. However, its optimum role is not established; therefore, decision-making should be individualized. Lipoprotein apheresis (LA) is another extracorporeal technique used for selective removal of lipoprotein particles from the blood with the return of the remaining components. It is indicated for lipoprotein (a) hyperlipoproteinemia (category II) [17].

Although several reports describe successful conservative management without TPE, normalization of triglyceride levels often required one to two months. Because our patient developed severe DKA complicated by AKI, followed by pneumonia, the initiation of TPE was associated with rapid clinical improvement. Although pancreatitis and cerebral edema were not established in this case, these too need prompt intervention and lowering of lipid status. There are no clear guidelines regarding the role of TPE in patients with uncontrolled diabetes complicated by DKA and severe combined hyperlipidemia. In such life-threatening situations, individualized management and timely aggressive intervention may improve clinical outcomes.

## Conclusions

DKA with severe combined hyperlipidemia as the first presenting feature of diabetes mellitus is relatively rare in the literature, particularly in the young age group. Our case was complicated by pneumonia and AKI. Early initiation of TPE was associated with rapid reduction of triglyceride levels, clinical improvement, and successful early liberation from life support. This case highlights the importance of a multidisciplinary approach in the management of complex DKA with severe hypertriglyceridemia and associated complications. Although TPE may be a valuable therapeutic option in selected patients, its high cost and limited availability in resource-constrained settings such as Bangladesh may restrict its routine use. Additional clinical experience and larger studies are needed to better define the role and indications of TPE in similar cases. This report adds to the limited body of literature and may contribute to informing future clinical decision-making.
